# Clustering properties of the cardiac ryanodine receptor in health and heart failure

**DOI:** 10.1016/j.yjmcc.2023.10.012

**Published:** 2023-10-27

**Authors:** Helen M.M. Waddell, Valeria Mereacre, Francisco J. Alvarado, Michelle L. Munro

**Affiliations:** a Department of Physiology and HeartOtago, School of Biomedical Sciences, University of Otago, Dunedin, New Zealand; b Department of Medicine and Cardiovascular Research Center, University of Wisconsin-Madison School of Medicine and Public Health, Madison, WI, USA

**Keywords:** Ryanodine receptor, Heart failure, Nanoscale remodelling, Clusters

## Abstract

The cardiac ryanodine receptor (RyR2) is an intracellular Ca^2+^ release channel vital for the function of the heart. Physiologically, RyR2 is triggered to release Ca^2+^ from the sarcoplasmic reticulum (SR) which enables cardiac contraction; however, spontaneous Ca^2+^ leak from RyR2 has been implicated in the pathophysiology of heart failure (HF). RyR2 channels have been well documented to assemble into clusters within the SR membrane, with the organisation of RyR2 clusters recently gaining interest as a mechanism by which the occurrence of pathological Ca^2+^ leak is regulated, including in HF. In this review, we explain the terminology relating to key nanoscale RyR2 clustering properties as both single clusters and functionally grouped Ca^2+^ release units, with a focus on the advancements in super-resolution imaging approaches which have enabled the detailed study of cluster organisation. Further, we discuss proposed mechanisms for modulating RyR2 channel organisation and the debate regarding the potential impact of cluster organisation on Ca^2+^ leak activity. Finally, recent experimental evidence investigating the nanoscale remodelling and functional alterations of RyR2 clusters in HF is discussed with consideration of the clinical implications.

## 1. Overview

The ryanodine receptor type 2 (RyR2) is a Ca^2+^ release channel located on the sarcoplasmic reticulum (SR) membrane of cardiac myocytes, with the appropriate release of SR Ca^2+^ via RyR2 being a key determinant of cardiac function. RyR2 is a large homo-tetrameric ion channel with a total molecular weight of ~2.2 MDa. The overall shape of RyR2 resembles a mushroom, with the stem embedded in the SR membrane and the ~30 nm diameter cap [1] protruding into the cytosol. An RyR2 tetramer forms the functional ion channel; however, channels are further assembled into clusters within the SR membrane. The organisation of these clusters, and their remodelling on a nanoscale, has recently drawn interest as a mechanism for regulating RyR2 function, which may be altered in cardiac diseases, particularly heart failure (HF). Understanding the key terminology in nanoscale RyR2 cluster descriptors is essential to the interpretation of cluster remodelling. However, a comprehensive explanation of these terms is currently lacking from existing literature. In this review, we provide a guide on RyR2 cluster organisational parameters, and the associated terminology used in this field. Discussion of the evidence for how changes in these parameters influence RyR2 Ca^2+^ handling properties and proposed mechanisms involved in cluster remodelling within the working myocardium is detailed. We also describe the application of super-resolution imaging approaches which have begun to elucidate these mechanisms. Furthermore, we discuss the recent experimental evidence for the role of nanoscale RyR2 cluster remodelling as an underlying mechanism of impaired Ca^2+^ handling in cardiac pathology, with a focus on HF.

## 2. RyR2-mediated Ca^2+^ handling in cardiac myocytes

### 2.1. Excitation-contraction coupling

The ability of the heart to pump blood relies on the generation of contractile force by the myocardium. Excitation-contraction (EC) coupling is the process underlying this function, which requires the controlled cycling of Ca^2+^ within the cardiomyocytes [2]. An action potential generated by the sinoatrial node propagates through the myocardium to depolarise the sarcolemma. This activates the voltage-gated L-type Ca^2+^ channel (LTCC) to allow a small influx of Ca^2+^ into the cytosol (*I*_CaL_). RyR2 is activated by *I*_CaL_ in a process called Ca^2+^-induced Ca^2+^ release (CICR), triggering Ca^2+^ release from the SR [3]. The resulting increase of cytosolic [Ca^2+^], called a Ca^2+^ transient, enables myofilament crossbridge cycling for force production, and subsequent contraction of the heart [2]. Relaxation occurs when Ca^2+^ is removed from the cytosol, mainly by the electrogenic Na^+^-Ca^2+^ exchanger (NCX1) and the SR Ca^2+^ ATPase (SERCA2a) [4].

**Fig. 1.**
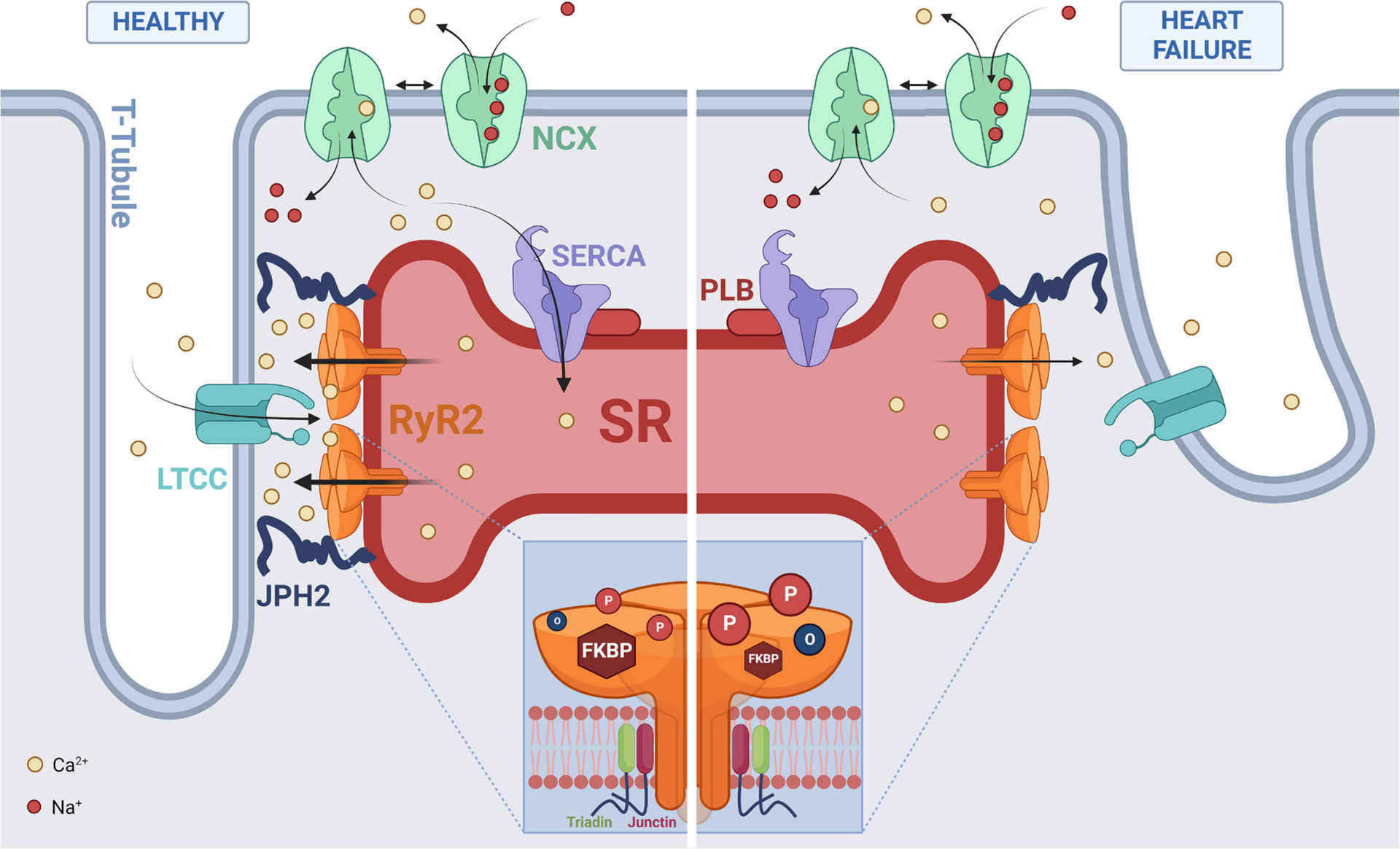
Cellular and RyR2 regulatory changes in HF. Schematic demonstrating the physiological organisation and regulation of ryanodine receptor (RyR2) in healthy cardiomyocytes (left) and changes associated with heart failure (HF; right). RyR2 is expressed in the sarcoplasmic reticulum (SR) membrane where it localises to the dyad with the L-Type Ca^2+^ channel (LTCC), facilitated by junctophilin-2 (JPH2). The Na^+^-Ca^2+^ exchanger (NCX) together with SR Ca^2+^-ATPase (SERCA; which is regulated by phospholamban (PLB)) remove cytosolic Ca^2+^ from LTCC influx and RyR2 release. Failing cardiomyocytes exhibit dilation, loss and disorganisation of t-tubules, along with reduced JPH2 expression leading to dyad disruption and separation of RyR2 from LTCC. Inset shows RyR2 demonstrates enhanced phosphorylation by PKA and CaMKII (‘P’ in red circle) and oxidation (‘O’ in blue circle), with reduced FKBP (maroon hexagon) association in failing hearts. Created with BioRender.com.

The efficiency of CICR is enhanced by the dyad, a microdomain formed by the direct apposition of the sarcolemma and junctional SR (SR_J_) membranes, which contain LTCC and RyR2, respectively (see Fig. 1). These regions are also called couplons or junctions [4]. To further enhance CICR and facilitate EC coupling, the sarcolemma forms invaginations called transverse (t-) tubules, enabling the formation of dyads within the cell interior. Peripheral couplings refer to the dyads formed at the surface sarcolemma, while those formed with the t-tubules are called internal couplings [5]. RyR2 clusters are distributed in a regular pattern along the z-disk of cardiomyocytes, aligned with the t-tubules which are spaced every ~2 μm along the long axis of the cell [6]. This organisation allows for the rapid, synchronised activation of RyR2 during EC coupling to produce a highly co-ordinated Ca^2+^ transient throughout the cardiomyocyte, which is essential for generating a strong cardiac contraction.

Spontaneous activation of RyR2 clusters is normal in the healthy myocardium because of the finite open probability of individual RyR2 channels [7]. This results in a low-level leak of Ca^2+^ out of the SR. Ca^2+^ leak occurs in the form of Ca^2+^ sparks (Ca^2+^ release events from RyR2 clusters that that can be visualised using fluorescence microscopy [8]) and as ‘silent’ or ‘invisible’ leak. Ca^2+^ released via ‘silent’ or ‘invisible’ leak has a lower amplitude and dissipates faster, such that it cannot be detected as visible Ca^2+^ sparks [9,10]. The cellular Ca^2+^ transient produced during EC coupling results from the temporal and spatial summation of Ca^2+^ sparks throughout the cardiomyocyte, synchronised by coordinated *I*_CaL_ and CICR activity.

### 2.2. RyR2 Ca^2+^ handling alterations in heart failure

HF is a multifactorial syndrome in which the heart is unable to pump sufficient blood to the systemic circulation [11]. HF can arise a result of acquired conditions (such as myocardial infarction) or secondary to inherited diseases that progressively damage the cardiac muscle (such as arrhythmogenic cardiomyopathy). Hence, the molecular mechanisms leading to HF are complex and sometimes unique (see review [12] for details). Systolic HF (also known as HF with reduced ejection fraction; HFrEF, and the focus of this review), involves weak cardiac contractions associated with perturbations of cellular Ca^2+^ homeostasis [13,14]. Diastolic HF (or HF with preserved ejection fraction; HFpEF) has recently gained attention as a separate clinical entity. The unique mechanisms underlying HFpEF are beyond the scope of this review. Characteristic Ca^2+^ transient changes in HF include slower release kinetics, a reduced Ca^2+^ transient amplitude and cardiomyocyte-wide dyssynchrony in Ca^2+^ release [14–19]. These perturbations are tightly linked to altered RyR2 regulation and function.

In HF, Ca^2+^ release via RyR2 is significantly increased during diastole. The resulting enhanced and persistent pathological Ca^2+^ leak may deplete the SR store, leading to a reduced transient amplitude and impaired contraction. Numerous animal and human HF studies report increased RyR2 activity which confers enhanced sensitivity to activation by Ca^2+^, further promoting Ca^2+^ leak, observed by either increased spark frequency, single channel opening probability, or silent Ca^2+^ release [20–23]. It is generally considered that increased RyR2-mediated Ca^2+^ leak is a significant contributor to SR load depletion, and consequently, the weaker contractions characteristic of HF.

In addition to cytosolic Ca^2+^, RyR2 is also sensitive to Ca^2+^ levels within the SR. Ca^2+^ leak is enhanced with elevated SR Ca^2+^ levels or when RyR2 becomes over-sensitised to cytosolic or luminal Ca^2+^ [24]. Hence, Ca^2+^ sparks may occur due to high SR Ca^2+^ levels and are also referred to as ‘store overload-induced Ca^2+^ release’ (SOICR) [25]. It is unknown if SOICR also underlies ‘silent’ Ca^2+^ leak as it is associated with lower SR Ca^2+^ levels [10]. What determines whether Ca^2+^ leak will be a spark or ‘silent’ is poorly understood. However, it is well documented that t-tubules are lost in systolic HF (reviewed extensively elsewhere [26–28]). The occurrence of Ca^2+^ sparks relies on the presence of t-tubules [29] since the spatial constraints of the dyadic cleft reduce the diffusion of leaked Ca^2+^ and in turn increase spark fidelity (likelihood of occurrence). Therefore, the propensity of ‘silent’ Ca^2+^ leak in HF is likely accelerated by t-tubule loss. Enhanced SR Ca^2+^ leak in HF is also detrimental to the electrical stability of the heart. Sensitised RyR2 channels are more prone to release Ca^2+^. Therefore, leak that would normally manifest as invisible leak may trigger full sparks, while leak that would appear as isolated sparks may activate neighbouring Ca^2+^-release sites and propagate throughout the cell as a Ca^2+^ wave. This spontaneously released Ca^2+^ is extruded from the cell by NCX1 leading to depolarisation of the sarcolemma, known as delayed after-depolarisations (DADs) [30]. When DADs are of a large enough magnitude, they can lead to spontaneous action potentials and cardiac arrhythmia [31].

## 3. Regulation of RyR2 function

### 3.1. Modulation via modifications and accessory proteins

Due to a combination of its large size and the bulk of the channel being located cytosolically within the dyadic cleft, RyR2 demonstrates multiple post-translational modifications and protein-protein interactions which can regulate channel function. RyR2 can undergo phosphorylation and redox modifications, with phosphorylation being the most extensively studied regulator of RyR2 function. Kinases regulating RyR2 include protein kinases A and G (PKA and PKG, respectively) and Ca^2+^/calmodulin-dependent protein kinase II (CaMKII). At least three sites are targeted by these kinases, which are collectively known to phosphorylate S2030, S2808 and S2814 to increase channel activity [32–34] (for review see [35]). Recently, phosphorylation of RyR2 by striated muscle preferentially expressed protein kinase (SPEG) at site S2367 has been demonstrated to have an inhibitory effect on leak activity [36]. Modification of RyR2 through oxidation has been shown to activate the channel in a biphasic manner [20,37]. Dephosphorylation of RyR2 has a similar effect on channel function [38,39]; however, this phenomenon and its physiological implications are not fully understood.

RyR2 forms a macromolecular complex with a number of known binding partners, including junctophilin (JPH2), FK506-binding proteins (FKBP), sorcin, triadin, junctin, calmodulin (CaM), as well as protein phosphatases PP1 and PP2A [40]. JPH2 spans the dyadic cleft and interacts with both SR_J_ and t-tubular membranes, ensuring RyR2 clusters remain in close apposition to t-tubules. Importantly, JPH2 also stabilises RyR2 to reduce Ca^2+^ leak [41,42]. Similarly, FKBP stabilises RyR2 and reduces channel activity when bound to the cytosolic face of RyR2 [43,44].

### 3.2. Altered RyR2 regulation in heart failure

In failing hearts, RyR2 exhibits hyperactivity due to changes in post-translational modifications and protein interactions (summarised in Fig. 1), which collectively increase diastolic Ca^2+^ leak. Hyperphosphorylation of RyR2 by PKA and CaMKII in HF has been associated with increased Ca^2+^ leak and an impaired Ca^2+^ transient [18,45,46]. Oxidative stress is also elevated in HF, which increases oxidation of free thiols within RyR2 and promotes Ca^2+^ leak [20,37,47]. Commonly, oxidation of RyR2 and hyperphosphorylation are observed together in HF and likely have an additive effect [18,20]. While RyR2 phosphorylation in HF has been extensively researched, it has not been without controversy. It was initially proposed that phosphorylation at S2808 (pS2808) by PKA caused dissociation of FKBP12.6, which in turn lead to increased RyR2 activity [46]. Marx and colleagues went on to show that preventing pS2808 mitigated HF progression in mice following myocardial infarction. However, others could not replicate these results [48,49]. The role of CaMKII phosphorylation at S2814 (pS2814) is clearer. The consensus in the field is that this modification increases Ca^2+^ leak in different forms of heart disease [50]. Studies on the role of S2030 in HF lag, but recent publications suggest that increased phosphorylation of this site by PKA also occurs in heart disease [51]. This debate remains ongoing and is reviewed extensively elsewhere [52].

Unlike phosphorylation and FKBP association, the role of JPH2 in HF has more consensus in the scientific community, with reduced JPH2 expression in rodent HF models associated with t-tubule loss and enhanced Ca^2+^ leak [53–55]. However, it is unclear whether this finding translates to humans, since there is discrepancy whether JPH2 loss occurs in HF patients [56,57]. This indicates that the role of JPH2 in HF is likely dependent on the etiology of the specific patient group and warrants further investigation.

## 4. Nanoscale RyR2 cluster parameters

**Fig. 2.**
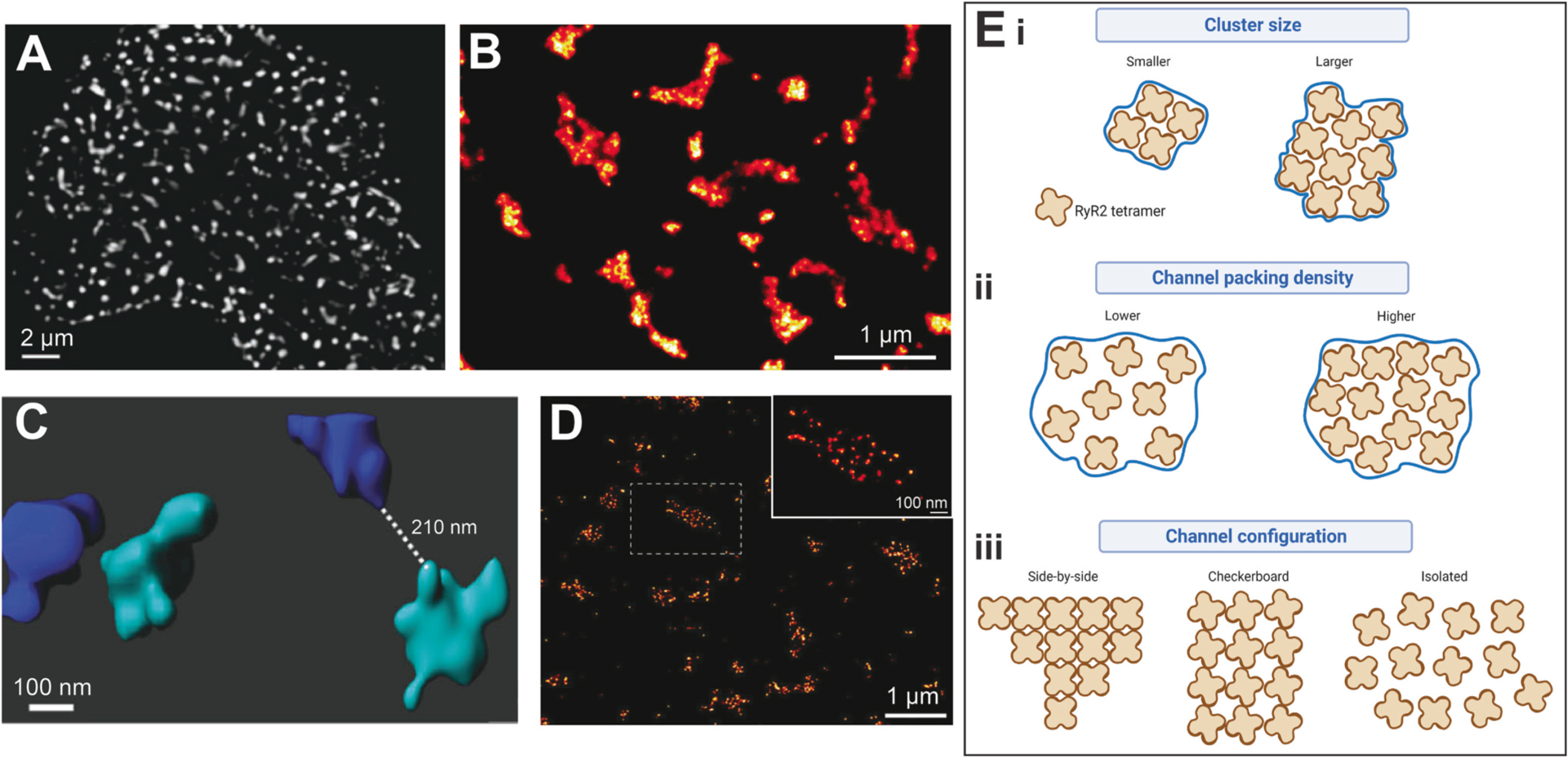
Individual RyR2 nanoscale clustering parameters. Example images of RyR2 clusters in non-failing rat ventricular cardiomyocytes visualised using A) confocal, B) dSTORM, C) 3D dSTORM and D) DNA-PAINT imaging modalities, demonstrating differences in image resolution. E) Schematic demonstrating changes in individual RyR2 cluster parameters associated with i) cluster size, ii) RyR2 channel packing density within a cluster, and iii) different arrangement of RyR2 tetramers within a cluster, showing side-by-side, checkerboard and isolated configurations. Blue line represents the outline edge of a single RyR2 cluster. Panel A modified from Soeller et al., 2009 [118], image reproduced with permission; Panel B modified from Hou et al., 2015 [65], image reproduced with permission; Panel C modified from Shen et al., 2019 [68]; Panel D modified from Jayasinghe et al., 2018 [66]. Panel E created with BioRender.com.

The first reported observation that RyR2 channels assemble into clusters within the SR membrane came from electron microscopy (EM) studies in the 1990s [58]. Since then, numerous investigations have described the properties of these clusters using a variety of imaging tools, each with its own set of advantages and limitations. For example, while EM provides high spatial resolution to distinguish individual RyR2 tetramers, the nature of sample preparation typically results in a small region being imaged at an unknown orientation (relative to the whole myocyte). This can make it difficult to determine the representative nature of the clusters imaged compared the cell-wide population, while also potentially influencing the accuracy of size measurements [59]. The advent of fluorescent imaging using protein tags or antibodies conjugated to fluorophores enabled whole cell and tissue samples to be examined, providing wider context of the findings. However, traditional fluorescent microscopy techniques which are limited by the diffraction of light, such as confocal microscopy, have a spatial resolution limit of ~250 nm [60]. This is significantly larger than the reported size of a single RyR2 tetramer (~370 Å diameter [61]), meaning that reported cluster sizes and remodelling changes may be underappreciated using these approaches (Fig. 2A). The development of super-resolution imaging techniques (e.g. direct stochastic optical reconstruction microscopy (dSTORM), photo-activatable localisation microscopy (PALM) and stimulated emission depletion (STED)) has enabled this diffraction limit to be bypassed (Fig. 2B–D), providing <30 nm spatial resolution to be achieved in relatively large biological samples [62]. However, this technique is not without disadvantages, including high temporal and computational requirements for data collection and processing (for review see [63]).

When characterising RyR2 clusters, there are several key parameters which are commonly assessed. These range from the nanoscale properties of individual channels, including cluster size and intra-cluster channel organisation, through to the distribution of clusters in relation to each other or key subcellular structures, such as the t-tubules or z-disk. Interpreting the role of these parameters in Ca^2+^ handling and cardiac function can be complex, as there is currently no single unified reporting method to enable direct comparisons between studies.

### 4.1. Cluster size

One of the most widely reported RyR2 cluster parameters is individual cluster size (Figure 2Ei). The reported mean cluster size in cardiomyocytes varies, ranging between 7 and 267 tetramers [6,41,58,64–68]. This disparity appears to be influenced by several factors, including the species and chamber examined, peripheral versus internal clusters, as well as the experimental imaging modality used. With advances in imaging techniques which provide increasing improvements in spatial resolution, the more recently reported size of an ‘average’ RyR2 cluster has decreased compared to earlier studies. It became evident that what was being reported as single clusters in confocal imaging studies were in fact close groupings of multiple small, individual clusters [64]. The majority of recent super-resolution studies are in general agreement, reporting means of ~9–19 RyR2 channels per cluster in non-diseased ventricular myocytes of both rats and humans [21,43,57,66,68]. It has also been demonstrated that not only is there large heterogeneity in sizes of RyR2 cluster present within a single sample, but a high proportion of these clusters are very small, with ~25–56% of clusters containing a single RyR2 tetramer [65,68]. It should be noted that it is currently unclear what the maximum separating distance is to allow neighbouring channels to still be considered as “clustered”, with most studies relying on changes in fluorescence intensity to distinguish individual clusters from one another.

Cluster size has traditionally been reported as the number of individual RyR2 channels that can fit within a cluster. This number is calculated using the reported tetramer centre-to-centre spacing of ~30 nm (900 nm^2^ area per channel). However, this conversion is based on the assumption that RyR2 channels demonstrate isotropic packing within each cluster, in which channels are aligned in a quasi-crystalline array, or ‘checkerboard’ manner [65,69]. It has since been demonstrated that this is not the case, with distinct separation observed between tetramers within a cluster [70], which is occupied by other dyadic proteins such as JPH2 [66]. This has led to some studies opting to report RyR2 cluster size as the unconverted unit area as a more representative measurement. Furthermore, this observation has highlighted the importance of understanding how channels are arranged within clusters when interpreting the impact on Ca^2+^ handling properties.

### 4.2. Channel packing density and configuration

Many original assumptions on RyR2 clustering properties were based the behaviour of RyR1, the skeletal muscle isoform of the channel, which has been observed to form clusters by assembling into lattices with a regular ‘checkerboard’ arrangement [69]. However, it is now acknowledged that RyR2 tetramers are not uniformly organised, but instead exist with varying channel densities and configurations within clusters in cardiomyocytes. With first generation super-resolution imaging modalities such as dSTORM, relative changes in the density of RyR2 channels packed within each cluster can be inferred from the intensity of the pixels representing a rendered cluster (which itself is proportional to photo-switching event counts and the underlying protein density) [71]. This is often termed ‘RyR2 density’ or ‘packing density’ and typically has arbitrary units [67] (Figure 2Eii). While this approach enables relative changes in channel packing density to be determined within individual studies, it is not suitable for comparisons between different studies. This is due to differences in experimental protocols which impact the rate and detection of single molecule photo-switching events (such as fluorophore selection, mounting medium, laser properties, camera). These differences therefore influence the final image rendering and pixel intensity, meaning that only relative changes within each individual experimental paradigm are valid comparisons. However, the application of 3D-dSTORM (Fig. 2C) has highlighted the need to take care when interpreting RyR2 localisation data, as discrete clusters in nearby focal planes may appear as overlapping when imaged as 2D data. This can lead to the over-estimation of RyR2 cluster size, as well as confound channel density measurements [68].

While super-resolution techniques, such as dSTORM and STED can provide ~30–60 nm resolution to infer channel density [64,72], newer approaches are able to resolve structures even further (e.g. DNA-PAINT, [Fig. 2D] and EM tomography). Using these approaches, it has become possible to not only determine the exact number of tetramers contained within an individual RyR2 cluster, but also measure the distance separating those channels. Studies utilising these techniques report the spacing between adjacent channels within a cluster as ranging between ~27–56 nm [43,66,73], confirming the variable nature of RyR2 tetramer organisation within clusters. This advancement from first generation super-resolution imaging enables a more robust and comparable measurement of intra-cluster RyR2 channel packing density.

Sophisticated EM approaches also enable the configuration of channels relative to each other to be visualised. It has been demonstrated that RyR2 tetramers can exist in checkerboard, side-by-side or isolated configurations (Figure 2Eiii), with clusters typically demonstrating a mixture of these configurations [43,70]. The exact configuration of channels is dynamic and can be modulated by regulatory factors such as phosphorylation and FKBP binding (discussed below). The shift between configurations is associated with a change in the spacing distance between tetramers as the channels redistribute within a cluster. Tetramers in a side-by-side configuration demonstrate closer spacing to one another (~31 nm) compared to checkerboard (~37 nm) or isolated configurations (~42 nm) [43,70].

### 4.3. Regulation of individual RyR2 clusters and implications for Ca^2+^ handling

Cluster size and intra-cluster channel arrangement have been proposed as key regulators of RyR2 channel function. However, there are conflicting theories regarding the relationship between RyR2 cluster organisation parameters and channel function, particularly in the context of Ca^2+^ leak activity. This largely arises from an ongoing debate as to how channels physically and functionally interact each other within clusters.

When considering cluster size, on one hand it has been proposed that smaller clusters, which contain a lower number of RyR2 tetramers, have fewer stabilising interactions between neighbouring channels. This is suggested to increase open probability and subsequently result in enhanced diastolic leak [74,75]. This mechanism would also apply to altered channel density within a cluster, whereby an increased spacing between channels (reduced channel density) would limit the formation of these interactions to promote leak activity. Similarly, an increase in channel density has been suggested to lead to steric hinderance between the more densely packed tetramers, resulting in reduced leak [43]. In this scenario, neighbouring tetramers would physically impair the spontaneous opening of each other [70]. Both of these mechanisms would result in a reduction in channel open probability, and thus lower Ca^2+^ leak activity. Recent experimental evidence supports this theory, whereby a smaller inter-channel distance (increased packing density) is associated with reduced Ca^2+^ leak activity [43]; although it should be noted these structure and function experiments were performed in parallel, rather than direct correlation.

Conversely, recent experimental work by Galice et al. revealed that smaller RyR2 clusters (<70 channels) demonstrate reduced Ca^2+^ leak occurrence compared to larger clusters [76]. One explanation for this observation is that if every RyR2 channel has a given open probability, the presence of more channels within a cluster increases the overall likelihood that the cluster as a whole will exhibit leak; thereby, smaller clusters would have a lower propensity to generate a spark. It should be noted, however, that this latter evidence was collected using diffraction-limited imaging methods, which are not able to resolve very small clusters [64], such as those previously associated with diastolic leak activity [74]. In addition, it has been proposed that smaller RyR2 clusters are more prone to demonstrate silent leak [9,21], limiting the assumptions on the relationship between cluster size and Ca^2+^ leak when measured by sparks. Furthermore, the characterisation of clusters as ‘small’ (<70 channels) is relative, considering the criterion by Galice et al. included clusters up to ~3 to 7-fold larger than recently reported mean sizes in super-resolution imaging studies. Further studies also suggest that the distribution of cluster sizes is an important determinant of leak propagation, with higher heterogeneity of cluster size within a cardiomyocyte associated with increased Ca^2+^ wave generation [77].

An additional concept of inter-channel regulation is that there is cooperative gating between RyR2 tetramers. It has been suggested that the opening of one RyR2 channel facilitates a conformational change in neighbouring channels through a physically interaction (potentially facilitated by FKBP), promoting the opening of additional channels within the cluster to trigger a spark [78,79]. In this scenario, an increase in RyR2 channel packing density would promote these interactions, which would enhance Ca^2+^ leak probability. Another version of cooperative gating is the presence of ‘local control’, in which Ca^2+^ leak through one channel within a cluster can trigger CICR of neighbouring channels, thus generating a Ca^2+^ spark [80]. Again, this would be promoted by an increased density (reduced spacing) of RyR2 tetramers.

Studies examining mechanisms which regulate the organisation of RyR2 channels within clusters have provided some additional insights, with phosphorylation and FKBP binding identified as two key factors. Alignment to a predominantly checkboard configuration is observed with high levels of RyR2 phosphorylation, while FKBP (12 or 12.6) binding drives channels towards a side-by-side arrangement [43,70]. In agreement with previous reports, functionally, these changes are associated with an increase in Ca^2+^ spark frequency with phosphorylation (checkerboard), and a decreased spark frequency with enhanced FKBP binding (side-by-side) in parallel Ca^2+^ imaging experiments [43]. This may be, at least in part, due to changes in regulatory channel-channel interactions, as described above, with side-by-side configuration conferring increased steric hindrance to reduce leak, while checkboard arrangements could facilitate cooperative gating mechanisms to enhance Ca^2+^ leak. We hypothesise that an isolated configuration would reduce spark fidelity and enhance silent leak due to reduced cooperative gating with the increased channel separation. It should be noted that these changes in channel configuration are also associated with changes in overall cluster size; in particular, smaller clusters are observed with high FKBP binding [43]. Additional computation modelling studies suggest that the shape of an RyR2 cluster significantly influences Ca^2+^ release dynamics. Elongated or irregular cluster shapes demonstrate a lower spark fidelity, with increased ‘silent’ leak profiles compared to clusters with the same number of tetramers present in a regular (circular or square) distribution [9,21,81]. Thus, it has become apparent that both cluster size and intra-cluster channel organisation are implicated in regulating RyR2 function.

The disparity in the interpretation of the effect of RyR2 cluster remodelling on leak is difficult to consolidate with the current lack of direct correlative data between Ca^2+^ handling properties and highly resolved nanoscale structure. The ability to perform both live-cell Ca^2+^ imaging and super-resolution imaging in the same subset of RyR2 clusters is technically challenging, limiting our current understanding of this fundamental relationship. Studies performing parallel structure and function experiments, and those examining remodelling changes in cardiac pathologies, including HF, provide some insights to this structure-function dynamic.

### 4.4. Cluster density, nearest neighbour distance and Ca^2+^ release units

**Fig. 3.**
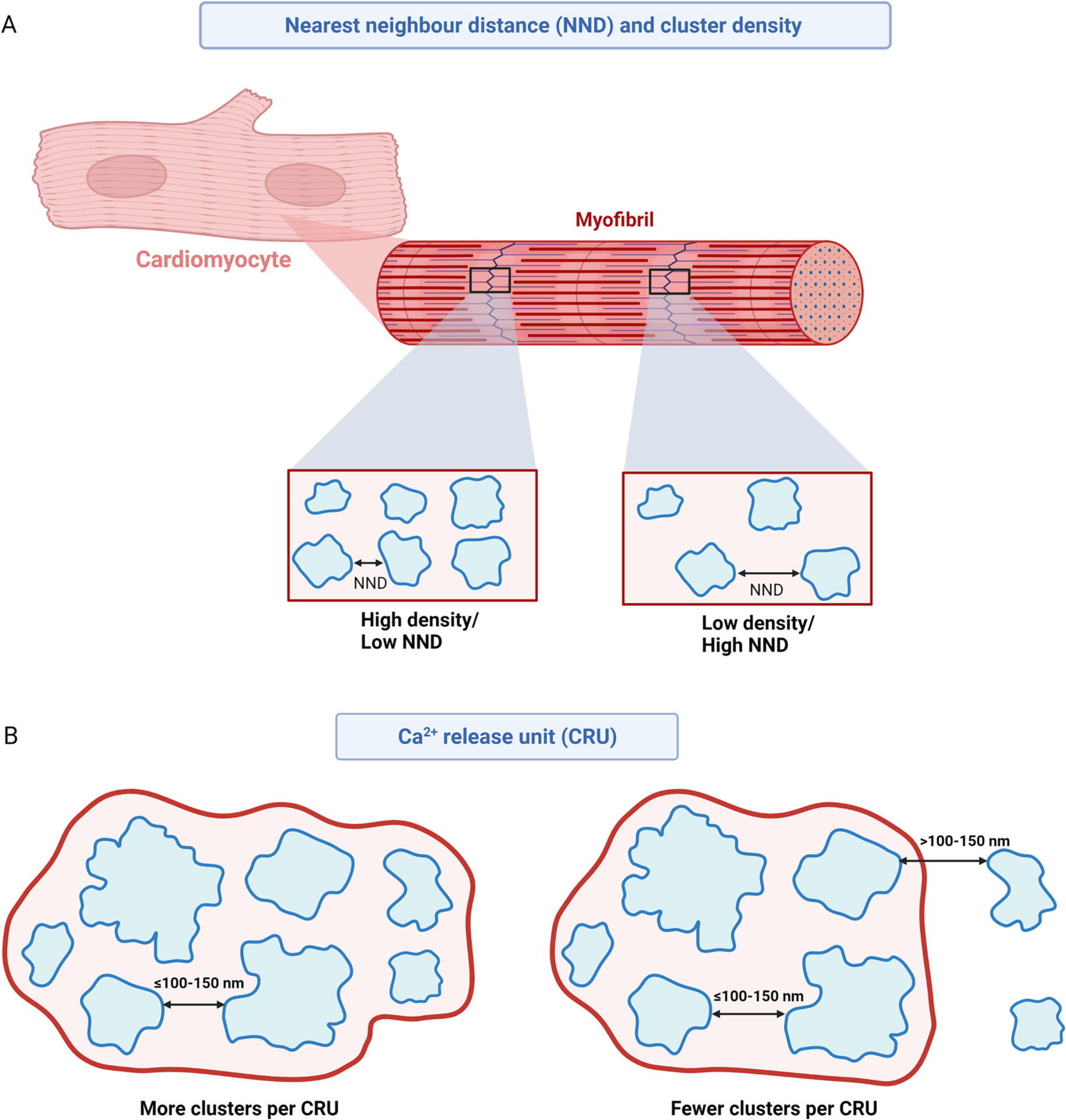
Inter-cluster parameters. Schematic demonstrating changes in RyR2 inter-cluster parameters. A) Representation of RyR2 distribution to the z-disk, demonstrating changes in RyR2 cluster density within a cardiomyocyte and the relationship with nearest neighbour distance (NND) Blue outline represents a single RyR2 cluster. B) The functional grouping of RyR2 clusters into a Ca^2+^ release unit (CRU; red boundary) when separated by NND of <100–150 nm. An increase in NND reduces the number of clusters per CRU, while clusters separated by >100–150 nm are excluded from the CRU. Created with BioRender.com.

In addition to individual cluster parameters, it is being increasingly recognised that the organisation of RyR2 clusters in relation to each other plays a critical role in Ca^2+^ handling properties in cardiac physiology and pathophysiology. Therefore, many groups investigating RyR2 nanoscale organisation also assess inter-cluster properties. Two of the fundamental inter-cluster parameters commonly assessed are cluster density and the inter-cluster nearest neighbour distance (NND) (Fig. 3A). Cluster density refers to the number of individual clusters contained per unit area or volume of the cardiomyocyte, while NND is the separation between one individual cluster and its closest neighbouring cluster (typically reported as an edge-to-edge distance, although some studies utilise centre-to-centre measurements). Logically, cluster density and NND are inversely related, whereby an increased number of clusters within a set area will reduce the mean spacing between those clusters. 2D and 3D cluster density measurements range between 2.2 clusters per μm^3^ and 4.5 clusters per μm^2^ in cardiomyocytes from healthy hearts [21,65]. Recently reported values of edge-to-edge NND in non-diseased cardiomyocytes fall between 140 and 164 nm [65,68], while centre-to-centre measurements are larger at 215–380 nm [21,72].

Unlike the controversies surrounding individual cluster properties discussed above, there is general agreement on the functional impact of altering these inter-cluster parameters. It is widely accepted through experimental and modelling-based evidence that a reduction in inter-cluster NND (an increase in cluster density) promotes the propagation of Ca^2+^ leak through the cardiomyocyte [72]. Considering that RyR2 is a Ca^2+^-sensitive Ca^2+^ release channel, there is potential for the Ca^2+^ released by one cluster (e.g., a Ca^2+^ spark) to trigger CICR in neighbouring clusters. A shorter NND increases the likelihood that the Ca^2+^ released by one cluster will be of a sufficient concentration to trigger CICR in the adjacent cluster, and thus propagate the Ca^2+^ release. This mechanism has been shown to contribute to arrhythmogenesis in a sheep model of atrial fibrillation (AF) [72].

The concept that neighbouring RyR2 clusters can be functionally coupled in this manner also led to the description of Ca^2+^ release units (CRUs; also termed ‘super-clusters’ in some studies [41,65]), in which sufficiently close neighbouring clusters are functionally grouped together as a unit capable of co-operative Ca^2+^ release [21,72]. It should be noted that some early studies examining RyR2 organisation used ‘CRU’ as the terminology to describe the assembly of Ca^2+^ handling proteins involved in EC coupling within a specialised domain (i.e., LTCC and RyR2 within a dyad) [58], which can cause some confusion for those becoming familiar with this field. However, it is now widely accepted that a CRU describes a group of RyR2 clusters which have the potential to be functionally coupled through CICR (Fig. 3B).

The most commonly reported parameter relating to CRUs is the number of individual RyR2 clusters present, with mean values reported to be 2.1–3.7 clusters per CRU in non-diseased cardiomyocytes [21,65,68,72]. Interestingly, recent 3D super-resolution imaging work by Shen et al., revealed that approximately half of all CRUs contain only a single RyR2 cluster [68]. This suggests that the prevalence of ‘rogue’ clusters may have been under-recognised in previous studies due to limitations of two-dimensional imaging techniques (for more details see [68]). In addition to cluster counts, many studies also report the number of RyR2 channels contained with the CRU. This vastly varies between studies, with means of ~18–103 tetramers per CRU reported [21,65,68,72]. However, this typically relies on calculating how many RyR2 tetramers can fit within the measured cluster area(s) of each CRU, as described previously for single clusters, and as such is subject to the same limitations regarding assumptions of channel packing within a cluster.

While there is consistency in the recent use of terminology relating to CRUs, the exact properties which define a CRU can vary between studies. This discrepancy is largely attributed to differences in the distance which is considered sufficiently small enough to allow the diffusion of a Ca^2+^ spark to trigger CICR in a neighbouring cluster. Based on different modelling approaches, this distance is estimated to be ~100–150 nm within a dyadic cleft [72,74]. Subsequently, studies have used varying distances within this range as their criterion for identifying the clusters that form a single CRU. Kolstad et al., compared the resulting CRU characteristics when applying either a 100 nm or 150 nm criterion. Unsurprisingly, the use of the larger CRU inclusion distance demonstrated an increase in the number of clusters and RyR2 tetramers contained per CRU [21]. Despite differences in the exact parameters of CRU formation, there is consensus on the functional impact of CRU remodelling within a cardiomyocyte. Modelling studies consistently identify that the presence of more individual clusters per CRU increases the likelihood of pathological Ca^2+^ leak activity [21,72]. This is not surprising considering that an increase in the number of clusters within a CRU is associated with a reduction in NND, and the aforementioned mechanism of propagating CICR between neighbouring clusters.

## 5. RyR2 cluster nanoscale remodelling in heart failure

**Fig. 4.**
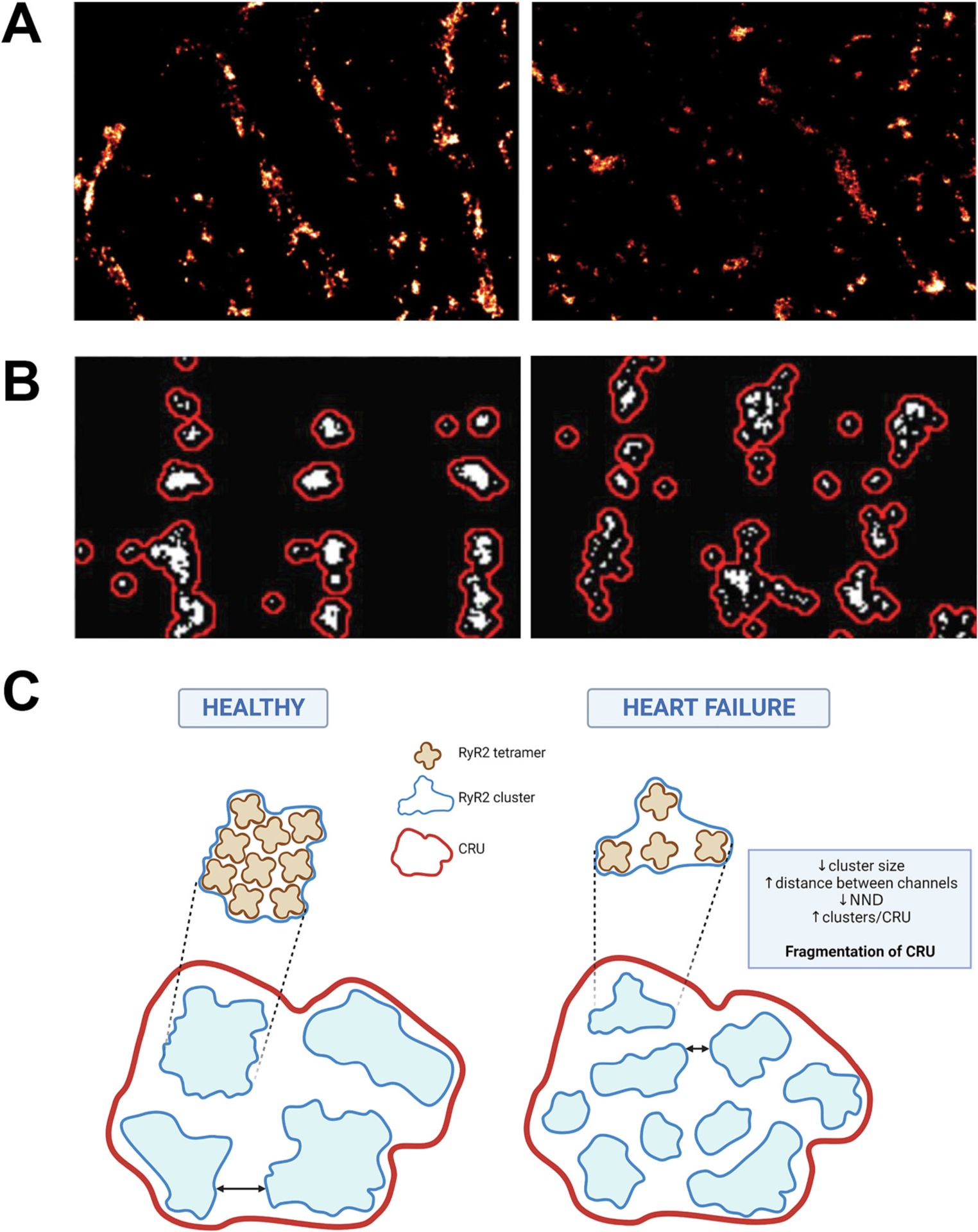
Nanoscale RyR2 cluster remodelling in heart failure. A) dSTORM images of RyR2 clusters in cardiac tissue from non-failing patients (left) and patients with end-stage heart failure (HF; right). B) dSTORM images of RyR2 clusters and CRUs in cardiac myocytes from control (left) and HF model (right) rats. C) Schematic summarising the organisation of RyR2 clusters in healthy cardiomyocytes (left) and the reported cluster remodelling in human and animal models of HF (right). Clusters in failing hearts demonstrate a reduction size and an increased distance between channels within a cluster (lower channel packing density). Inter-cluster nearest neighbour distance (NND) is reduced, with a higher cluster density and increased number of individual clusters (blue outline) per Ca^2+^ release unit (CRU; red boundary) in failing cardiomyocytes. Panel A images modified from Hou et al., 2021 [57]. Panel B images modified from Kolstad et al., 2018 [21]. Panel C created with BioRender.com.

The nanoscale properties of RyR2 clusters have been studied for over a decade with the focus recently shifted towards understanding the role of cluster remodelling as a mechanism in cardiac pathogenesis, including HF. Here, we discuss recent findings in this field and the potential implications, with experimental evidence and a proposed summary of changes presented in Fig. 4.

### 5.1. Individual cluster remodelling

As previously described, size is a fundamental property of RyR2 clusters and is therefore one of the most widely reported cluster parameters when assessing nanoscale cluster remodelling. In a coronary artery ligation model of HF in rats, clusters were found to be ~33% smaller compared to sham controls [21], with similar changes observed using expansion microscopy in a rat model of right-side HF [73]. This was coupled with an increase in the distance between channels within a cluster in HF animals, suggesting reduced channel packing density [73]. This remodelling is often referred to as cluster ‘fragmentation’. Hou et al., describe a trend towards reduced cluster size in the ventricle of patients with end-stage HF from idiopathic dilated cardiomyopathy (IDCM), however this was not statistically significant [57]. Interestingly, individual RyR2 clusters are reportedly unchanged in other cardiac pathologies associated with increased Ca^2+^ leak activity, namely AF [67,72]. These findings suggest that while cluster size is likely implicated in HF-associated Ca^2+^ dysfunction, additional factors are at play which contribute to the pathogenesis of different cardiac diseases.

One of the most widely investigated factors in RyR2 regulation is phosphorylation. Hyper-phosphorylation of RyR2 is commonly reported in HF samples, as well as in some forms of AF. As previously described, high levels of RyR2 phosphorylation can trigger channel reconfiguration within clusters, as well as changes in cluster size. Using a ‘phosphorylation cocktail’ to activate both PKA and CaMKII results in a subtle increase in overall cluster size, with enhanced RyR2 phosphorylation at S2814 (pS2814) [43,70]. However, experimental evidence points towards a reduction in RyR2 cluster size in HF samples, associated with chronic, pathologically driven phosphorylation. This discrepancy may be explained by differences in mechanisms underlying artificially driven acute phosphorylation (e.g., phosphorylation cocktail or isoproterenol treatment) compared to chronic hyper-phosphorylation and additional subcellular remodelling observed in HF.

When examining the distribution of phosphorylated RyR2 channels within a cluster, Sheard et al., demonstrated that there is a uniform distribution of a low proportion of pS2808 residues in control cells. When stimulated with isoproterenol, this proportion increased, while still displaying a uniform pattern throughout the cluster [73]. Interestingly, examination of HF clusters revealed a distinct pattern, with pS2808 residues predominantly located towards the centre of the cluster [73]. Modelling demonstrated that this cluster profile is more prone to generate sub-spark, or silent, Ca^2+^ leak activity, compared to high spark fidelity in clusters with acute phosphorylation profiles [73]. However, other findings suggest that central phosphorylation domains within a cluster increase spark fidelity compared to uniform distributions [81]. The conflicting findings of these studies are likely due to differences in the distribution of RyR2 tetramers within a cluster used in each model, which as previously discussed, can significantly impact channel gating properties. However, both studies propose that the increase in RyR2 phosphorylation is a compensatory mechanism activated to attempt to restore Ca^2+^ handling dynamics which are impaired in HF [73,81]. This further suggests that acute and chronic phosphorylation of RyR2 may contribute to differences in both function and structural alterations in physiological and pathological settings. To test this theory, Shen et al., compared the effect of acute and chronic phosphorylation on cluster organisation using a combination of pharmacological and genetic approaches. β-adrenergic stimulation was observed to cause a progressive reduction in interior RyR2 cluster size following prolonged exposure but had no effect on sub-surface clusters [82]. Similar changes were identified when either CaMKII or PKA were selectively activated. Transgenic mice which mimic permanent CaMKII phosphorylation of RyR2 demonstrated similar remodelling to prolonged isoproterenol treatment, while mice unable to be phosphorylated at this residue still display cluster remodelling following adrenergic stimulation [82]. These findings indicate that both CaMKII and PKA are involved in pathological remodelling of RyR2 clusters similar to that observed in HF models.

Another regulatory factor to which may influence cluster organisation in HF is the presence of FKBP. As previously described, enhanced FKBP binding decreases RyR2 cluster size with tetramer reconfiguration [43]. It should be noted that FKBP was not actively dissociated from control cells in this study, so a basal level of binding would be expected in these clusters. Given the controversies surrounding ‘healthy’ FKBP occupancy of RyR2 and its role as a regulator of RyR2 function, it is difficult to hypothesise on the potential impact of reducing FKBP association with RyR2, as is proposed by some groups to occur in HF (for review see [52]). To our knowledge, the effect of altered FKBP binding properties on nanoscale RyR2 clustering parameters has not been directly assessed in the setting of HF.

The presence of additional proteins within the RyR2 cluster is also a consideration in assessing dyadic and cluster remodelling, particularly JPH2. Several studies report down-regulation of JPH2 in failing or arrhythmic hearts [42,54,56,83,84]; however, this is not a unanimous finding [57,85]. JPH2 has been shown to co-cluster with RyR2 with a high degree of co-localisation and homology in the shape of the two protein co-clusters [86]. In failing IDCM patient hearts, there is no difference observed in the extent of RyR2-JPH2 co-localisation compared to donor hearts [57]. However, in an animal model of HF there is a ~ 45% loss of JPH2 from the dyad, with the remaining JPH2 observed to predominantly localise to the centre of RyR2 clusters [87]. This is proposed to leave the cluster periphery less stable and more prone to undergo remodelling, with RyR2 appearing more scattered at the cluster edges [87]. Transgenic mice with induced loss of JPH2 expression develop HF with t-tubule disruption and enhanced Ca^2+^ leak activity, while demonstrating unchanged RyR2 cluster size or channel packing density [41,55]. Conversely, JPH2 over-expressing mice demonstrate enlarged RyR2 clusters with a reduction in channel density within each cluster [41]. While previous evidence would suggest that this increased separation between RyR2 channels should result in enhanced Ca^2+^ leak propensity, these mice demonstrate a reduction in both Ca^2+^ spark size and frequency. The ~3-fold increased ratio of JPH2 relative to RyR2 is suggested to enhance channel stability, thus reducing the occurrence of leak compared to control mice [41]. These findings indicate that the presence of additional dyadic proteins, such as JPH2, is an additional factor which can influence both the organisation and function of RyR2 clusters, and its role in HF pathophysiology is likely dependent on mechanisms underlying different disease etiologies.

### 5.2. Cluster distribution alterations

The distribution of RyR2 clusters in relation to one another determines the potential of CRU formation and the properties of Ca^2+^ leak propagation. Kolstad et al., were the first to describe a reduction in NND between RyR2 clusters in a rat model of HF, with a corresponding increase in cluster density. This inter-cluster remodelling was associated with an increase in the number of clusters per CRU, leading to the description of CRU ‘fragmentation’ in HF [21]. When coupled with the reduced cluster size observed in this HF model, the result is an overall loss of RyR2 tetramers from the CRU [21]. Modelling experiments confirm that this pattern of CRU fragmentation drives a pathological phenotype of prolonged Ca^2+^ leak of low amplitude, consistent with the enhanced silent leak activity observed in HF [21]. Similar CRU remodelling has also been observed in a sheep model of AF [72] (although not in AF patients [67]), demonstrating a potential role of RyR2 clustering in driving arrhythmogenesis.

In contrast, a larger NND is reported in failing IDCM patients [57]. Surprisingly, this does not significantly alter CRU formation, despite a reduction in both the number of clusters and RyR2 tetramers per unit area of cell. This was attributed to a ~ 50% reduction in RyR2 expression in failing patients [57]. In a rabbit model with 50% RyR2 knockdown, a similar overall reduction in cluster density was also observed without overt changes to cellular Ca^2+^ handling and cardiac function [88]. This was associated with smaller, more densely packed clusters which demonstrated an increased prevalence of forming large, multi-cluster CRUs [88]. These findings suggest that reduced RyR2 expression is a key driver of nanoscale remodelling for both individual clusters and inter-cluster organisation. However, the exact phenotype of this remodelling appears to be dependent on the mechanism underlying the loss of protein expression: failing hearts exhibit profound structural and electrophysiological remodelling [57], unlike the rabbit model with genetic depletion of RyR2 [88].

Utilising direct correlative Ca^2+^ imaging and PALM super-resolution imaging in transgenic mice expressing photo-activatable RyR2, Hou et al., have provided the first ground-breaking insights into the Ca^2+^ release properties of super-resolved RyR2 clusters. The authors have provided experimental evidence that sparks can be generated from underlying single or multi-release events [89]. Single release events are more likely to originate from smaller clusters, while multi-release sparks are associated with larger clusters and increased RyR2 density. This is also the first experimental evidence to demonstrate the propagation of Ca^2+^ leak as multi-release sparks between neighbouring channels and clusters [89]. It should be noted that these data were generated from sub-sarcolemmal clusters which may have different structural and functional dynamics compared to internal dyads [82]. A model of HF generated in these mice was observed to have enhanced Ca^2+^ leak propagation, with prolonged Ca^2+^ sparks observed, despite a similar spark frequency to control animals [89]. Furthermore, the RyR2 nanoscale remodelling driving this functional impairment was described to be similar to the reported CRU dispersion in rat HF models, with reduced inter-cluster spacing between smaller clusters [89].

## 6. Clinical implications

The role of RyR2 cluster remodelling in driving pathological Ca^2+^ handling is becoming a recognised mechanism underlying cardiac dysfunction in HF. Importantly, similarities in cluster remodelling are observed in models of failure and arrhythmia, as detailed above, both of which are associated with increased Ca^2+^ leak via RyR2. While in HF, the increased leak is well described as contributing to the reduced Ca^2+^ transient and contractile dysfunction observed, it can also trigger the development of DADs. This is a common mechanism for arrhythmogenesis in HF and other syndromes. This is particularly relevant as HF is associated with an increased susceptibility to arrhythmia, with sudden cardiac death due to lethal arrhythmia occurring in ~50% of HF patients [90].

Despite the widely acknowledged role of RyR2-mediated Ca^2+^ leak in HF, there are currently few treatment options which appropriately target this mechanism clinically. This is largely due to difficulties in maintaining the balance between targeting RyR2 channels to block pathological leak, while still allowing CICR-mediated activity. At present, one of the primary pharmacological interventions for HF patients is the prescription of β-blockers (e.g., carvedilol) [91]. Mechanistically, this reduces the work of the heart by lowering heart rate and contractility, in part by preventing PKA-mediated phosphorylation of RyR2 (among other targets). However, despite this treatment, hyperphosphorylation of RyR2 at S2814 is observed in HF patients [92], which has implications for cluster remodelling (as discussed above). As such, additional targeting of CaMKII-mediated pathways represents a potential avenue for reducing RyR2 cluster remodelling and Ca^2+^ mis-handling in HF.

Although not currently clinically approved for HF treatment, selective RyR2-targeting drugs provide an interesting prospect for preventing Ca^2+^ leak in failing hearts and other ryanopathies, including arrhythmia. Dantrolene is one such example. Traditionally used as an RyR1 antagonist to block Ca^2+^ leak in malignant hyperthermia, dantrolene has also shown the potential to target RyR2 for the prevention of cardiac arrhythmogenesis. Studies in animal models of HF and arrhythmia reveal the anti-arrhythmic properties of dantrolene through a reduction in Ca^2+^ leak activity [93,94] and pRyR2 levels [95]. This anti-arrhythmic effect has recently been recapitulated in human cardiac samples [96]. Interestingly, the ability of dantrolene to supress RyR2 channel opening has been demonstrated to rely on the binding of both CaM and FKBP12.6 [97,98]. The role of FKBP12.6 binding is also implicated in RyR2-targeting compounds known collectively as Rycals. In particular, Rycals JTV519 and S107 have been identified as promoting stabilisation of RyR2-FKBP12.6 binding to reduce RyR2 open probability and prevent subsequent arrhythmic activity [99–102]; however, the role of FKBP12.6 binding in this effect has been debated [103]. A clinical trial exploring a second generation Rycal, ARM210, for targeting RyR2-mediated arrhythmia is currently planned [104]. Verticilide, a derivative of a fungal compound, is another example if a RyR2 regulator that shows therapeutic potential. Verticilide inhibits hyper-active RyR2 and has shown antiarrhythmic potential in a model of inherited arrhythmia [105], AF [106] and arrhythmogenic cardiomyopathy [107]. Given the high incidence of arrhythmia in HF patients [90], dantrolene, Rycals and verticilide present promising therapeutic avenues to explore. Whether the therapeutic mechanisms of these drugs have implications for regulating RyR2 cluster organisation remains to be determined.

## 7. Future directions and conclusions

To date, the research investigating RyR2 cluster remodelling as a mechanism in HF has focused on HFrEF. However, it is widely acknowledged that HFpEF is an equally prevalent form of HF which demonstrates distinct clinical characteristics and pathogenesis mechanisms [108]. Evidently, while t-tubule loss and disorganisation are commonly described forms of remodelling that occur in HFrEF [109–111], recent work by Frisk et al., has revealed that is not the case of HFpEF. Remarkably, t-tubule density was found to be increased in ventricular cardiomyocytes from HFpEF patients [16]. Further investigation of different rat HFpEF models also revealed that this densification appeared to be dependent on the presence of other co-morbidities, including hypertension or diabetes [16]. This indicates that subcellular remodelling mechanisms underlying different forms of HF can vary, thus warranting investigation to whether this is also true for RyR2 cluster changes. There is also evidence to suggest that the mechanisms and remodelling dynamics of dyads and RyR2 clusters is dependent on the localisation within the cardiomyocyte. It has been shown that clusters associated with t-tubules (internal) demonstrate enhanced JPH2 co-localisation compared to clusters found at the surface sarcolemma (peripheral couplings) or corbular SR (non-dyadic) [57]. Furthermore, there are significant differences in the size and NNDs of clusters within the different SR regions, independent of the presence of HF [57].

While the organisation of RyR2 clusters has a clear impact on Ca^2+^ handling properties and cardiac function, there is a dynamic interplay of the mechanisms underlying this remodelling with those driving additional changes in dyadic organisation. This includes the organisation of the t-tubule network, as well as the localisation of other key EC coupling proteins within the dyad in close proximity to RyR2. One example is altered JPH2 expression, whereby JPH2 loss is not only associated t-tubule disorganisation and reduced stability of RyR2 channels [55], but also results in reduced co-localisation of RyR2 and NCX1 to further impact Ca^2+^ signalling [112]. Similarly, bridging integrator 1 (BIN1) is implicated in regulating dyadic organisation through playing a key role in t-tubule development and anchoring LTCC within dyadic microdomains [113,114]. More recently, BIN1 has also been associated with recruitment of phosphorylated RyR2 into these microdomains [115], while a loss of BIN1 is observed in HF and is associated with disruption of the t-tubule network and impaired trafficking of LTCC [85,116]. It is therefore likely that mechanisms driving t-tubule and dyadic remodelling, such as altered BIN1 expression, will also impact RyR2 cluster organisation and co-localisation between dyadic proteins, and are of high interest to investigate further in the context of HF.

The continual development of improvements to imaging techniques provides additional opportunities for exploring these critical questions in cardiac pathologies. This includes the recent application of 3D dSTORM and correlative super-resolution imaging to elucidate not only the nanoscale organisation of RyR2 clusters, but also their detailed coupling to t-tubules in the formation of dyads, as well as the functional consequence of cluster remodelling with Ca^2+^ leak activity. Excitingly, recent reports of ~3 nm imaging resolution methodology provide the promise of even further resolved RyR2 clusters for understanding the implications of RyR2 cluster remodelling in health and disease [117].

Combined, we have reviewed evidence demonstrating RyR2 cluster organisation to be an important regular of channel function. The nanoscale properties of RyR2 clusters can be dynamically modulated in both physiological and pathological settings to influence Ca^2+^ leak activity. Importantly, the remodelling observed is a contributor to the impaired Ca^2+^ transient and reduced contractile function in HF. The ability to manipulate RyR2 cluster nanoscale organisation to regulate Ca^2+^ leak occurrence represents an intriguing potential avenue to pursue in the development of novel therapies for cardiac pathologies, in particular HF.
